# Outcome of Blunt Abdominal Traumas with Stable Hemodynamic and Positive FAST Findings 

**Published:** 2016

**Authors:** Firooz Behboodi, Zahra Mohtasham-Amiri, Navid Masjedi, Reza Shojaie, Peyman Sadri

**Affiliations:** 1 Department of General Surgery, Guilan University of Medical Sciences, Rasht, IR Iran.; 2 Department of Preventive & Social Medicine, Guilan University of Medical Science, Rasht, IR Iran.; 3 Department of General Surgery, Zanjan University of Medical Science, Zanjan, IR Iran.

**Keywords:** Abdominal injuries, wounds, nonpenetrating, patient outcome assessment, ultrasonography, tomography, X-ray computed

## Abstract

**Introduction:**

Focused assessment with sonography for trauma (FAST) is a highly effective first screening tool for initial classification of abdominal trauma patients. The present study was designed to evaluate the outcome of patients with blunt abdominal trauma and positive FAST findings.

**Methods:**

The present prospective cross-sectional study was done on patients over 7 years old with normal abdominal examination, positive FAST findings, and available abdominopelvic computed tomography (CT) scan findings. The frequency of need for laparotomy as well as its probable risk factors were calculated.

**Results:**

180 patients were enrolled (mean age: 28.0 ± 11.5 years; 76.7% male). FAST findings were confirmed by abdominopelvic CT scan in only 124 (68.9%) cases. Finally, 12 (6.6%) patients needed laparotomy. Mean age of those in need of laparotomy was significantly higher than others (36.75 ± 11.37 versus 27.34 ± 11.37, p = 0.006). Higher grading of spleen (p = 0.001) and hepatic (p = 0.038) ruptures increased the probability of need for laparotomy.

**Conclusion:**

68.9% of the positive FAST findings in patients with blunt abdominal trauma and stable hemodynamics was confirmed by abdominopelvic CT scan and only 6.6% needed laparotomy. Simultaneous presence of free fluid and air in the abdominal area, old age, and higher grading o solid organ injuries were factors that had a significant correlation with need for laparotomy.

## Introduction:

Traumatic injuries are the major cause of mortality in the under 40 year old population and abdominal trauma is the third common trauma with a high rate of morbidity and mortality (1-3). Rapid diagnosis and treatment can decrease the rate of abdominal trauma related mortality, up to 50% (4). Immediate referral of the victim to a trauma center and timely diagnosis and treatment play a key role in improvement of patient outcome. When a patient with suspected blunt abdominal trauma is presented to emergency department (ED), the physicians look to determine the presence of intra-abdominal injuries and predict patient outcome (5). Focused assessment with sonography for trauma (FAST) is a highly effective first screening tool for initial classification of abdominal trauma patients (6). Yet, like all sonography techniques, its diagnostic value depends on the operator’s skill. Therefore, the findings of the studies are sometimes contradictory (7-9). The results of most existing studies show that FAST has average sensitivity and high specificity in detection of abdominal traumatic injuries (8, 10, 11). The effect of using this diagnostic test on improvement of patient management is still a matter of debate. The present study was designed to evaluate the outcome of patients with blunt abdominal trauma and positive FAST findings.

## Methods:

The present prospective cross-sectional study was done with the aim of evaluating the outcome of patients with blunt abdominal trauma and positive FAST results. 180 patients presented to the ED of Poursina Hospital, Rasht, Iran, during 2013 and 2014 were enrolled using convenience sampling. The protocol of the present study was approved by the Ethics Committee of Guilan University of Medical Sciences. All the researchers adhered to the principles of Helsinki Declaration. Inclusion criteria consisted of age over 7 years, normal abdominal examination and positive FAST findings. Hemodynamic unstability, abnormal abdominal examination, pregnancy, lost to follow-up were among the exclusion criteria. The patients underwent abdominal sonography by a trained (for 8 hours) senior emergency medicine resident and in case of positive FAST findings, abdominopelvic computed tomography (CT) scan with intravenous (IV) contrast was performed. Demographic data (age, sex) as well as sonography and CT scan findings, and need for laparotomy were recorded. Need for laparotomy was considered the final outcome of the present study. FAST was carried out using a SonoScape SSI-5500BW ultrasonography machine with a 3.5 – 5 MHz probe. CT scan was done using a Toshiba Asteion 16 slice scanner and interpreted by a radiologist blind to FAST and clinical findings. Positive FAST was defined as presence of solid organ injury or free abdominal fluid in at least one of the Morison's, spleno-renal or retro-vesical pouches.

The sample size was calculated to be 170 cases, considering the 20% prevalence of need for laparotomy in positive FAST cases (12), α = 0.05, and d = 0.06. Data were analyzed using STATA 11.0. Quantitative data were reported as mean and standard deviation (SD) and qualitative ones as frequency and percentage. Independent t-test was used to compare the average age and hemoglobin level of operated and non-operated patients. In addition, chi square or Fisher’s exact tests were used to compare frequency. In all analyses p < 0.05 was considered as significance level.

## Results:

**Figure 1:**
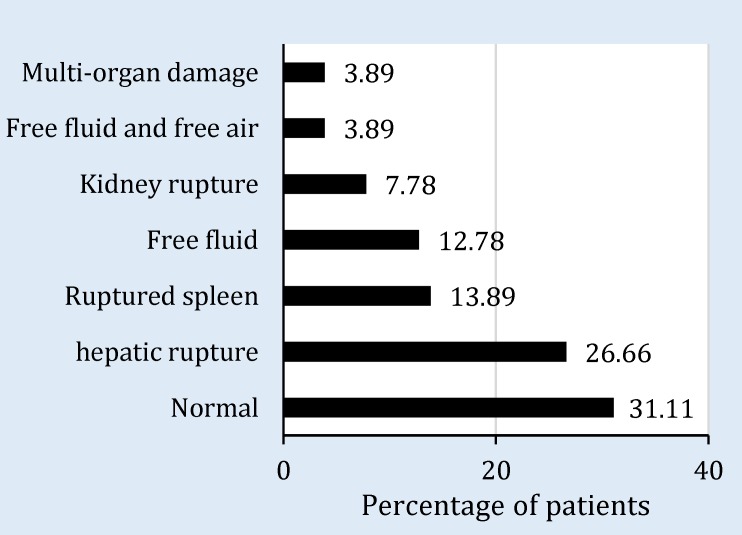
Abdominopelvic computed tomography scan findings of FAST positive patients

180 patients with blunt abdominal trauma, stable hemodynamic and positive FAST findings were enrolled (mean age: 28.0 ± 11.5 years; 76.7% male). Table 1 shows age and sex distribution of the participants. FAST findings were confirmed by abdominopelvic CT scan in only 124 (68.9%) cases (Figure 1). Hepatic (26.7%) and spleen (7.8%) ruptures, and presence of free fluid (12.8%) were the most common CT findings. Finally, 12 (6.6%) patients needed laparotomy, 7 (58.33%) of which had free abdominal fluid and air, 1 (8.33%) had ruptured liver, and 4 (33.34%) had ruptured spleen. Mean age of those in need of laparotomy was significantly higher than others (36.75 ± 11.37 versus 27.34 ± 11.37, p = 0.006). Higher grading of spleen (p = 0.001) and hepatic (p = 0.038) ruptures increased the probability of need for laparotomy (Table 2).

## Discussion:

Based on the results of the present study, only 68.9% of the positive FAST findings in patients with blunt abdominal trauma and stable hemodynamics was confirmed by abdominopelvic CT scan. Finally, only 6.6% of these patients needed laparotomy. Simultaneous presence of free fluid and air in the abdominal area, old age, and higher grading of solid intra-abdominal organ injuries were factors that had a significant correlation with need for laparotomy.

Cunningham et al. express that all patients with evidence of free abdominal fluid in CT scan need laparotomy (13). In a study by Yanar et al. only 33% of the patients needed laparotomy. They believe that non-operative treatment will be efficient in 75% of cases. This study showed that solid viscus score, more than 20% decrease in hematocrit, and level of serum lactate on admission are the most important predictive factors of non-operative management failure (14). The present study showed that simultaneous presence of free abdominal fluid and air is directly related to need for laparotomy. In addition, 93.3% of non-operative treatments were successful. This high rate might be due to the high percentage of solid organ injury cases, as a high percentage of solid organ injury patients, even those with higher grading, are successfully treated with non-operative treatments (15). The existing studies show that FAST has low sensitivity and high specificity in identifying traumatic abdominal injuries. Fox et al. showed that the presence of positive FAST findings is a proper predictor for presence of intra-abdominal injury (16). Additionally, Kelley et al. showed a 73% sensitivity and 100% specificity for FAST in identification of intra-abdominal traumatic injuries (17). In the present study, the number of false positive cases of FAST was high (31.1%). One of the reasons might be that accuracy of sonography has a significant correlation with the operator’s skill. Since in this study, FAST was performed by an emergency medicine resident, his skill and experience might have affected the interpretation of FAST data.

**Table 1:** Age and sex distribution of the patients

**Variable **	**Frequency (%)**
**Sex **	
Male	138 (76.7)
Female	42 (23.3)
**Age (years)**	
< 10	13 (7.2)
11-20	42 (23.3)
21-30	49 (27.2)
31-40	54 (30.0)
41-50	15 (8.3)
51-60	7 (3.9)

**Table 2:** The relationship of baseline characteristics and clinical findings with need for laparotomy

**Factors**	**Laparotomy**		**P**
	**Yes (n = 12)**	**No (n = 168)**	
**Age**	36.8±11.4	27.3±11.4	0.006
**Sex**			
Male	10 (83.3)	128 (76.2)	0.57
Female	2 (16.7)	40 (23.8)	
**Hemoglobin (mg/dl)**	13.0±1.8	12.2±1.7	0.12
**Grade of ruptured spleen**			
I	0 (0.0)	18 (64.3)	0.001
II	1 (25.0)	9 (32.1)	
III	1 (25.0)	1 (3.5)	
V	2 (50.0)	0 (0.0)	
**Grade of hepatic rupture**			
I	0 (0.0)	7 (13.7)	0.038
II	0 (0.0)	32 (62.8)	
III	0 (0.0)	11 (21.6)	
IV	1 (100.0)	1 (2.0)	
**Presence of air and fluid **			
No	5 (41.7)	168 (100.0)	<0.001
Yes	7 (58.3)	0 (0.0)	

One of the limitations of this study was its short follow-up duration. If long-term follow-up for 1, 6, or 12 months was done, factors affecting long-term outcome such as mortality or need for delayed surgery could be evaluated. Another limitation was performance of sonography by an emergency medicine resident. Although a sonography workshop was held for the resident, his lack of skill could affect the results.

## Conclusion:

Based on the results of the present study, only 68.9% of the positive FAST findings in patients with blunt abdominal trauma and stable hemodynamics was confirmed by abdominopelvic CT scan. Finally, only 6.6% of these patients needed laparotomy. Simultaneous presence of free fluid and air in the abdominal area, old age, and higher grading o solid organ injuries were factors that had a significant correlation with need for laparotomy.
